# Medication Adherence in Women with Early-Stage Breast Cancer and Active Parenting Responsibilities: The Mediating Role of Parenting Stress and Spiritual Well-Being

**DOI:** 10.3390/medicina62020306

**Published:** 2026-02-02

**Authors:** Veli Çakıcı, Aysel Oğuz, Süleyman Can, Gizem Bakır Kahveci, Hasibe Bilge Gür, Fahri Akgül, Abdurrahman Yiğit, Alper Topal, Pınar Peker, Erkan Özcan, İvo Gökmen, Yalçın Çırak

**Affiliations:** 1 Department of Internal Medicine, Division of Medical Oncology, Faculty of Medicine Hospital, Çanakkale Onsekiz Mart University, Çanakkale 17010, Türkiyeivo_georgiev1@hotmail.com (İ.G.);; 2 Department of Internal Medicine, Division of Medical Oncology, Faculty of Medicine Hospital, Necmettin Erbakan University, Konya 42080, Türkiye; 3 Department of Internal Medicine, Division of Medical Oncology, Faculty of Medicine Hospital, Trakya University, Edirne 22030, Türkiye; 4 Department of Internal Medicine, Division of Medical Oncology, Faculty of Medicine Hospital, Sakarya University, Sakarya 54000, Türkiye; 5 Department of Medical Oncology, Iğdır Dr. Nevruz Erez State Hospital, Iğdır 76000, Türkiye; 6 Department of Internal Medicine, Division of Medical Oncology, Mersin City Training and Research Hospital, Mersin 33010, Türkiye; 7 Department of Internal Medicine, Division of Medical Oncology, Faculty of Medicine Hospital, Tokat Gaziosmanpaşa University, Tokat 60100, Türkiye; 8 Department of Internal Medicine, Division of Medical Oncology, Adana City Training and Research Hospital, Adana 01060, Türkiye; 9 Department of Internal Medicine, Division of Medical Oncology, Kastamonu State Hospital, Kastamonu 37100, Türkiye

**Keywords:** breast cancer, medication adherence, parenting stress, spiritual well-being, psychosocial factors

## Abstract

*Background and Objectives*: Medication adherence is a key determinant of treatment effectiveness in early-stage breast cancer, particularly during long-term systemic therapies. As breast cancer is increasingly diagnosed at younger ages, a growing number of women continue to carry active parenting responsibilities during treatment. However, the associations between parenting-related psychosocial factors and medication adherence remain insufficiently explored. This study aimed to examine the associations between parenting stress, spiritual well-being, and medication adherence in women with early-stage breast cancer who maintain active parenting roles. *Materials and Methods*: This multicenter, cross-sectional study included 432 women with early-stage (I–III) breast cancer receiving active systemic therapy across nine oncology centers. Parenting stress was assessed using the Parenting Stress Scale (PSS), spiritual well-being using the Functional Assessment of Chronic Illness Therapy–Spiritual Well-Being Scale (FACIT-Sp-12), and medication adherence using the 6-item Modified Morisky Adherence Scale (MMAS-6). Spearman correlation analyses and multivariable linear regression models were used to evaluate associations between variables. Mediation analysis was performed using Hayes’ PROCESS macro (Model 4) with 5000 bootstrap samples to assess statistical mediation. *Results*: Parenting stress was positively associated with poorer medication adherence (ρ = 0.248, *p* < 0.01), whereas spiritual well-being was negatively associated with non-adherence (ρ = −0.225, *p* < 0.01). Parenting stress showed a strong inverse association with spiritual well-being (ρ = −0.597, *p* < 0.01). In multivariable regression analyses, both parenting stress and spiritual well-being were independently associated with medication adherence (β = 0.180, *p* = 0.002 and β = −0.199, *p* = 0.001, respectively). Mediation analysis demonstrated a significant indirect statistical association between parenting stress and medication adherence through spiritual well-being (indirect effect = 0.0155), consistent with partial statistical mediation. *Conclusions*: Medication adherence among women with early-stage breast cancer and active parenting responsibilities is associated with psychosocial context in addition to clinical factors. Parenting stress is associated with poorer adherence, whereas greater spiritual well-being is associated with better adherence within a statistical mediation framework. These findings generate hypotheses for future longitudinal and interventional studies.

## 1. Introduction

Breast cancer is the most commonly diagnosed malignancy among women, and advances in early detection and systemic treatments have led to substantial improvements in survival over recent decades [1]. Parallel to these advances, the age at diagnosis has shifted toward younger populations, resulting in a growing number of women who continue to carry active parenting responsibilities during and after treatment. For these patients, the cancer experience extends beyond biological disease processes and clinical interventions, unfolding within the broader psychosocial context of ongoing motherhood. Accordingly, evaluating treatment processes solely through clinical parameters may be insufficient to fully understand treatment adherence and patient engagement in this population [2].

Parenting stress refers to the persistent psychological strain associated with meeting a child’s physical, emotional, and developmental needs. In mothers diagnosed with breast cancer, this burden may be amplified by the concurrent demands of coping with illness-related stressors. Cancer treatments—including surgery, chemotherapy, radiotherapy, and endocrine therapy—are commonly associated with fatigue, body image disturbances, functional limitations, and emotional distress, which may exacerbate parenting stress and reduce quality of life [3,4]. Elevated parenting stress has been linked to guilt, perceived inadequacy, and emotional exhaustion, affecting both maternal psychological well-being and children’s emotional adjustment [5,6]. Despite these well-documented psychosocial effects, the extent to which parenting stress influences cancer-related health behaviors, particularly medication adherence, remains insufficiently explored [7,8].

The increasing use of oral anticancer therapies in clinical oncology has enhanced flexibility in treatment delivery and may improve quality of life. However, their effectiveness depends largely on patients’ consistent and accurate medication use. As responsibility for treatment administration shifts from healthcare providers to patients, adherence becomes particularly vulnerable in individuals experiencing substantial psychosocial burden. Women managing cancer treatment alongside active parenting responsibilities may therefore face unique challenges in maintaining optimal adherence, yet empirical evidence addressing this intersection remains limited [7,8].

Spiritual well-being represents an important psychosocial resource reflecting an individual’s capacity to derive meaning, maintain hope, and preserve inner balance in the face of adversity. Within psycho-oncology, higher spiritual well-being has been consistently associated with lower psychological distress, more adaptive coping strategies, and stronger motivation toward treatment engagement [9,10,11]. In this context, spiritual well-being may function as a protective factor buffering the negative impact of stress on health-related behaviors, including medication adherence.

Despite increasing attention to psychosocial factors in younger women with breast cancer, research on medication adherence has largely overlooked the specific impact of active parenting responsibilities. In particular, the mechanisms linking parenting-related stress to adherence behavior remain insufficiently explored. Although spiritual well-being has been associated with coping and quality of life in oncology populations, its potential mediating role in medication adherence has not been systematically examined. The present study addresses these gaps by focusing on women with early-stage breast cancer who continue to fulfill active parenting roles and by evaluating spiritual well-being as a potential statistical mediator in the association between parenting stress and medication adherence.

In this study, we examined the associations between parenting stress, spiritual well-being, and medication adherence in a large multicenter sample of women with early-stage breast cancer who continued to fulfill active parenting roles. These constructs were assessed using validated instruments, including the Parenting Stress Scale (PSS), the Functional Assessment of Chronic Illness Therapy–Spiritual Well-Being Scale (FACIT-Sp-12), and the 6-item Modified Morisky Adherence Scale (MMAS-6). Beyond direct associations, we specifically aimed to evaluate whether spiritual well-being mediates the relationship between parenting stress and medication adherence. By addressing this gap, the present study seeks to advance understanding of adherence behaviors within the lived psychosocial context of early-stage breast cancer.

## 2. Materials and Methods

### 2.1. Study Design and Participants

This multicenter, cross-sectional study was conducted between June and December 2025 across nine medical oncology centers in Türkiye. Women aged 18 years or older with a diagnosis of early-stage breast cancer (stage I–III) who were receiving active systemic therapy were consecutively recruited. Active systemic therapy included adjuvant or neoadjuvant chemotherapy, endocrine therapy, and/or oral systemic treatments administered with curative intent and participants were assessed during ongoing treatment at routine outpatient follow-up visits; no minimum or maximum duration of systemic therapy was required for eligibility. One of the key inclusion criteria was the presence of ongoing parenting responsibilities.

Active parenting was defined as having at least one child younger than 18 years of age or having a child aged 18 years or older who continued to live in the same household and for whom caregiving responsibilities were ongoing. The intensity of caregiving may still vary across participants (e.g., by the child’s age, number of dependents, and daily caregiving demands); subgroup/sensitivity analyses based on these dimensions were not pre-specified and are therefore considered in the interpretation of results. Patients with metastatic disease, those with cognitive impairment that precluded completion of self-report questionnaires, or severe psychiatric conditions interfering with reliable questionnaire completion, and those with incomplete data were excluded. A total of 432 patients who met the eligibility criteria were included in the final analyses.

### 2.2. Data Collection Procedure

Data collection was carried out using a standardized protocol across all participating centers by physicians trained in the study procedures. Eligible participants were approached during routine outpatient clinic visits and were provided with detailed verbal and written information regarding the study objectives, procedures, and use of data. Written informed consent was obtained from all participants prior to enrollment, both for participation in the study and for the processing and analysis of their personal data.

Questionnaires were administered in outpatient clinic settings under conditions designed to ensure participant comfort, privacy, and confidentiality. Assessments were completed in quiet and private areas, with sufficient time allocated (approximately 15–20 min) to minimize time pressure and external interruptions.

### 2.3. Measures

Medication adherence was assessed using the MMAS-6, with higher scores indicating poorer adherence to treatment. Spiritual well-being was measured using the FACIT-Sp-12, which assesses the domains of meaning, peace, and faith; higher scores reflect greater levels of spiritual well-being [12]. Parenting stress was evaluated using the PSS, with higher scores indicating greater levels of parenting-related stress [13].

Sociodemographic variables (including age, educational level, employment status, income level, household composition, and number of children) and clinical characteristics (including disease stage, time since diagnosis, treatment modalities, and number of medications used) were obtained from electronic medical records.

### 2.4. Statistical Analysis

Statistical analyses were performed using IBM SPSS Statistics for Windows, version 29.0. The distributional properties of continuous variables were assessed using the Shapiro–Wilk test. Nonparametric methods were applied when significant deviations from normality were observed. Comparisons between two groups were conducted using the Mann–Whitney U test, and comparisons among three or more groups were performed using the Kruskal–Wallis test. Associations between variables were examined using Spearman’s rank correlation coefficient.

Although the MMAS-6 is an ordinal scale and demonstrated non-normal distribution, total scores were treated as approximately continuous variables, as commonly accepted in adherence research, allowing the use of linear regression models to estimate associations between predictors and adherence outcomes. Multiple linear regression analyses were performed to identify independent predictors of medication adherence. Model assumptions, including multicollinearity, normality of residuals, and homogeneity of variance, were evaluated and confirmed. Variable selection for the multivariable and hierarchical regression models was based on a priori theoretical considerations rather than univariate screening. The primary aim of the regression analyses was to test a predefined psychosocial framework focusing on parenting stress and spiritual well-being as key constructs related to medication adherence. Other sociodemographic and clinical variables were therefore examined descriptively and through group comparisons to contextualize the findings but were not included in the final regression models to avoid overadjustment in this cross-sectional design.

In addition, mediation analysis was conducted to examine the mediating role of spiritual well-being in the relationship between parenting stress and medication adherence using Hayes’ PROCESS macro (Model 4) with 5000 bootstrap samples. Although mediation analysis is most robust in longitudinal designs, it can also be applied in cross-sectional studies to test theory-driven statistical pathways without implying causality. In this study, mediation analysis was used to examine whether the association between parenting stress and medication adherence could be statistically explained by variations in spiritual well-being. Bias-corrected 95% confidence intervals were calculated for indirect effects. Statistical significance was set at *p* < 0.05 for all analyses.

### 2.5. Ethical Considerations

The study was conducted in accordance with the principles of the Declaration of Helsinki. Ethical approval was obtained from the Çanakkale Onsekiz Mart University Non-Interventional Clinical Research Ethics Committee (Approval date: 18 June 2024; Decision No: 2025-09/09-44; Protocol No: 2025-178). Prior to study initiation, institutional permissions were obtained from all participating centers. Written informed consent was obtained from all participants before participation.

## 3. Results

A total of 432 women with early-stage breast cancer and active parenting responsibilities were included in the analysis. The study population exhibited substantial heterogeneity with respect to sociodemographic, caregiving-related, and clinical characteristics. (Table 1).

### 3.1. Descriptive Statistics and Associations Between Scales

The cohort predominantly consisted of women with stage II disease, most of whom lived in urban areas and reported sufficient or barely sufficient income; baseline sociodemographic, caregiving-related, and clinical characteristics are summarized in Table 1. The mean MMAS-6 score was 2.12 ± 1.36, the mean PSS score was 34.81 ± 10.64, and the mean FACIT-Sp-12 score was 34.77 ± 8.42. Median values and interquartile ranges indicated substantial heterogeneity in score distributions across all scales. Spearman correlation analysis demonstrated a positive and statistically significant association between MMAS-6 scores and parenting stress (ρ = 0.248, *p* < 0.01). In contrast, MMAS-6 scores were negatively associated with spiritual well-being (ρ = −0.225, *p* < 0.01). The strongest association was observed between PSS and FACIT-Sp-12 scores, revealing a moderate-to-strong inverse correlation (ρ = −0.597, *p* < 0.01) (Table 2).

### 3.2. Sociodemographic Characteristics and Scale Scores

Sociodemographic variables, including economic status, employment status, place of residence, and household composition, were significantly associated with MMAS-6, PSS, and FACIT-Sp-12 scores. Participants who reported sufficient income (53.0%) exhibited lower mean rank scores for MMAS-6 and PSS and higher mean rank scores for FACIT-Sp-12 (all *p* < 0.001). Conversely, participants with insufficient income (19.7%) demonstrated higher stress levels and poorer medication adherence.

Regarding place of residence, participants living in rural areas (14.4%) had significantly higher MMAS-6 and PSS scores compared with those residing in urban areas (70.4%) (both *p* < 0.001). The highest FACIT-Sp-12 scores were observed among participants living in semi-urban areas (*p* = 0.002). Participants living alone (4.6%) reported higher parenting stress and lower spiritual well-being compared with those living with others (both *p* < 0.001). While the number of children was not associated with MMAS-6 scores, an increasing number of children was significantly associated with higher PSS scores (*p* = 0.001) (Table 3).

### 3.3. Clinical Characteristics and Treatment Modalities

No significant associations were observed between time since diagnosis and MMAS-6, PSS, or FACIT-Sp-12 scores (*p* > 0.05). In contrast, disease stage and number of medications used were significantly associated with parenting stress and spiritual well-being. Participants with stage I disease (28.9%) exhibited lower PSS scores and higher FACIT-Sp-12 scores compared with those with more advanced stages (*p* < 0.01). Increasing numbers of medications were associated with higher stress levels and lower spiritual well-being, with the effect being particularly pronounced among patients using four or more medications (both *p* < 0.001).

With respect to treatment modalities, chemotherapy and surgery were not significantly associated with MMAS-6 scores. However, participants receiving radiotherapy had higher PSS scores (*p* = 0.006) and lower FACIT-Sp-12 scores (*p* = 0.017). Similarly, patients undergoing hormone therapy exhibited higher parenting stress and significantly lower spiritual well-being (both *p* < 0.001) (Table 4).

### 3.4. Medication Use and Adherence-Related Characteristics

The majority of participants (63.2%) reported taking their medications without using any reminder methods, while 20.8% indicated using reminders such as alarms or written notes. The most frequently reported barriers to medication use were treatment-related side effects (29.6%), forgetfulness (25.5%), and general difficulty in medication use (25.2%). The ability to report more than one barrier highlights the multifactorial nature of medication non-adherence in this population (Table 5).

### 3.5. Predictors of MMAS-6 Scores

Multiple linear regression analysis was performed to identify factors independently associated with MMAS-6 total scores. The overall model was statistically significant and explained 11.6% of the variance in MMAS-6 scores (R^2^ = 0.116; adjusted R^2^ = 0.112; F = 28.10; *p* < 0.001). Parenting stress emerged as a positive independent predictor of MMAS-6 scores (β = 0.180; *p* = 0.002), whereas spiritual well-being was identified as a negative independent predictor (β = −0.199; *p* = 0.001) (Table 6).

To evaluate the hierarchical contribution of these variables, a hierarchical regression analysis was conducted. In Model 1, parenting stress alone significantly predicted MMAS-6 scores (R^2^ = 0.091; F(1430) = 43.14; *p* < 0.001). The addition of FACIT-Sp-12 scores in Model 2 resulted in a significant increase in explained variance (ΔR^2^ = 0.025; ΔF(1429) = 11.96; *p* = 0.001). In the final model, both variables independently predicted MMAS-6 scores, and no evidence of multicollinearity was observed (VIF = 1.60) (Table 7).

**Figure 1:**
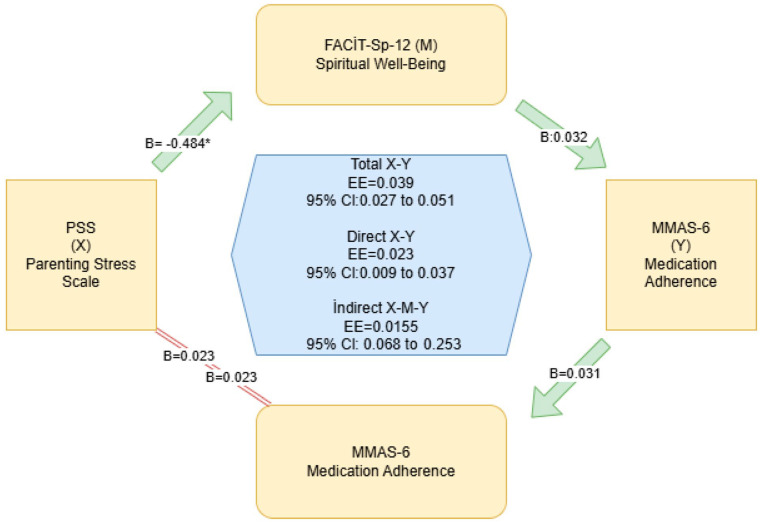
Mediation Analysis Demonstrating the Indirect Effect of Parenting Stress on Medication Adherence Through Spiritual Well-Being (Pathways represent statistical associations based on cross-sectional data and do not imply causality). * Statistically significant effect (*p* < 0.05).

In addition, mediation analysis was conducted to examine the mediating role of spiritual well-being in the relationship between parenting stress and medication adherence. Parenting stress was significantly associated with lower FACIT-Sp-12 scores (path a: B = −0.484; *p* < 0.001), and FACIT-Sp-12 scores were associated with lower MMAS-6 scores after controlling for parenting stress (path b: B = −0.032; *p* = 0.001). Both the direct effect (c′: B = 0.023; *p* = 0.002) and the total effect (c: B = 0.039; *p* < 0.001) were statistically significant. The indirect effect was also significant (a × b = 0.0155; 95% CI: 0.0068–0.0253), indicating partial mediation (Table 8 and Figure 1).

## 4. Discussion

In this large multicenter sample of women with early-stage breast cancer and active parenting responsibilities (n = 432), medication adherence (MMAS-6; higher scores indicating poorer adherence) was found to be associated with factors extending beyond clinical and disease-related characteristics alone. The observed associations between PSS, FACIT-Sp-12, and adherence behavior suggest that treatment adherence may be shaped by patients’ broader life context. These findings align with contemporary frameworks emphasizing that behavioral outcomes in cancer care—such as adherence, self-management, and treatment engagement—are influenced by psychosocial burden, caregiving roles, and available social resources, independent of disease severity [14,15,16].

The positive association between parenting stress and poorer adherence, alongside the inverse relationship between spiritual well-being and non-adherence, highlights the co-occurrence of vulnerability- and resilience-related psychosocial factors in this population. The strongest observed association between parenting stress and spiritual well-being indicates that higher caregiving-related stress levels tend to coincide with lower levels of meaning, inner balance, and existential resources. This pattern is consistent with prior systematic reviews demonstrating that higher spiritual well-being is associated with lower psychological distress and more adaptive coping in oncology populations [10,17]. Moreover, evidence supporting meaning-centered interventions suggests that strengthening existential resources is associated with improvements in emotional outcomes, symptom burden, and treatment engagement [18,19]. Accordingly, spiritual well-being may be conceptualized not merely as a dimension of belief, but as a psychosocial resource that supports patients’ capacity to navigate the cancer experience.

The persistence of these associations in multivariable models further indicates that parenting stress and spiritual well-being are independently associated with adherence behavior. Parenting stress emerged as a significant positive predictor of MMAS-6 scores, whereas spiritual well-being was independently associated with better adherence. Although the explained variance of the regression model was modest (R^2^ ≈ 0.12), this magnitude is consistent with adherence models in oncology, where treatment-related, cognitive, financial, emotional, and health system factors interact within a complex “treatment burden” framework [16,20]. This modest explanatory power likely reflects the absence of other well-established determinants of medication adherence, such as cognitive characteristics, health literacy, treatment-related beliefs, and the quality of clinician–patient communication, which were beyond the scope of the present model. Importantly, although the effect sizes observed were small, psychosocial factors such as parenting stress and spiritual well-being are potentially modifiable and may have meaningful clinical implications when targeted cumulatively or in combination with other adherence-enhancing interventions. Together, these findings underscore the multifactorial nature of medication adherence and support the integration of psychosocial dimensions into adherence-focused care models. Additional determinants of adherence, such as depression, anxiety, social support, health literacy, treatment perceptions, and clinician–patient communication, have been shown to contribute to adherence behavior and were not captured in the present model, which may partly explain the modest proportion of explained variance.

Hierarchical regression analyses further supported this interpretation. In Model 1, parenting stress alone accounted for 9.1% of the variance in MMAS-6 scores, whereas the inclusion of spiritual well-being in Model 2 increased the explained variance to 11.6%, representing a statistically significant improvement. This finding suggests that spiritual well-being contributes unique explanatory information regarding medication adherence beyond its shared variance with parenting stress. Prior studies demonstrating that depressive symptoms are associated with reduced adherence and that resilience-related constructs, such as empowerment, are associated with enhanced treatment engagement, provide a conceptual framework consistent with the protective association of FACIT-Sp-12 observed in the present study [15].

These statistical relationships were further explored through mediation analysis. Parenting stress was statistically associated with lower spiritual well-being, while lower spiritual well-being, in turn, was associated with poorer medication adherence after controlling for parenting stress. The presence of a significant partial indirect effect indicates a statistical mediation pattern, suggesting that the association between parenting stress and adherence may be partly accounted for by variations in spiritual well-being. Given the cross-sectional nature of the data, these findings should be interpreted as statistical associations rather than evidence of temporal or mechanistic pathways. From a clinical perspective, this pattern raises the possibility that interventions focusing exclusively on stress reduction may not fully address adherence-related challenges, and that approaches simultaneously supporting meaning, purpose, and spiritual well-being merit further investigation [15,21,22].

Sociodemographic and socioeconomic patterns further delineated areas of vulnerability within this population. Participants reporting insufficient income exhibited higher parenting stress, poorer adherence, and lower spiritual well-being compared with those reporting adequate income. Similarly, individuals residing in rural areas demonstrated higher stress and lower spiritual well-being than those living in urban settings, while participants living alone represented a particularly vulnerable subgroup with the highest stress levels and lowest spiritual well-being. These findings are consistent with global evidence linking financial toxicity and geographic or logistical barriers with increased psychosocial burden and compromised treatment-related behaviors in cancer care [23,24].

With respect to clinical variables, the absence of significant associations between time since diagnosis and PSS, FACIT-Sp-12, or MMAS-6 scores suggests that the passage of time alone may not be sufficient to alleviate stress or improve adherence. In contrast, a clear gradient was observed between disease stage and psychosocial burden: as disease stage increased, parenting stress rose and spiritual well-being declined. Notably, higher disease stage was not statistically associated with poorer adherence, supporting the notion that adherence behavior may be more closely related to contextual factors, such as daily life manageability, caregiving demands, and social support, than to biological disease severity alone [25,26]. These findings are consistent with reports from the PSYCHE study group, which demonstrated that parenting stress in mothers with breast cancer is more strongly associated with depressive symptoms and child-related factors than with clinical disease characteristics alone [6].

Treatment modalities further provided important contextual insight. Although the majority of participants received chemotherapy (91.4%) and surgery (97.2%), neither modality was statistically associated with medication adherence. In contrast, patients receiving radiotherapy exhibited higher parenting stress and lower spiritual well-being, and similar patterns were observed among those undergoing hormone therapy. These findings suggest an association between longer-term or sustained treatments and increased psychosocial burden, potentially related to cumulative side effects, ongoing treatment demands, and disruptions to daily routines. Accordingly, the concept of “treatment burden” appears particularly relevant in this population, highlighting the close interplay between sustained oncologic therapies and psychosocial functioning [27,28].

Self-reported reasons for non-adherence identified several actionable intervention targets. The majority of patients reported not using any reminder strategies for medication intake, while a smaller proportion relied on alarms, written notes, or family support. The most commonly reported barriers were treatment-related side effects, forgetfulness, and general difficulty with medication use, followed by daily fatigue and stress, and, less frequently, mistrust of medication. This distribution underscores the need to address medication adherence through multilevel strategies targeting biological, cognitive, and contextual factors simultaneously [29,30]. Moreover, higher non-adherence among patients serving as primary caregivers, along with increasing stress and declining spiritual well-being as childcare demands intensified, suggests that parenting responsibilities are associated with both practical and psychosocial challenges during treatment [6,31].

This multidimensional pattern has implications for future intervention research. While digital reminders and structured behavioral strategies have demonstrated efficacy in improving adherence, evidence suggests that such approaches may be insufficient in individuals experiencing high psychosocial burden [32]. Consistent with findings from the ROSETA pilot randomized trial, which highlighted the limitations of education-only interventions, our results support the hypothesis that psychosocially integrated adherence strategies warrant further evaluation [32]. In particular, patients facing socioeconomic disadvantage, rural residence, social isolation, primary caregiving responsibilities, polypharmacy, and prolonged adjuvant treatments may represent high-risk groups for whom early identification and referral to multicomponent support programs could be explored. This perspective aligns with contemporary oncology guidelines emphasizing psychosocial care as an integral component of comprehensive cancer treatment [33].

Key strengths of this study include its focus on a large and clinically relevant population of women with early-stage breast cancer who continue to fulfill active parenting roles, as well as the integration of correlation and regression findings with mediation analysis to examine the PSS → FACIT-Sp-12 → MMAS-6 pathway within a statistical modeling framework. This methodological approach is consistent with contemporary literature emphasizing the importance of pathway-based analyses in understanding health-related behaviors [34]. Nevertheless, several limitations should be acknowledged. The cross-sectional design precludes causal inference, and bidirectional relationships between variables cannot be excluded. In addition, the temporal ordering of parenting stress, spiritual well-being, and medication adherence cannot be confirmed. Accordingly, the mediation analysis should be interpreted as describing statistical associations rather than temporal or causal mechanisms. Medication adherence was assessed using a self-reported scale, which may be susceptible to recall bias and social desirability bias. The absence of objective adherence measures should be considered when interpreting the findings. In addition, the modest explanatory power of the regression model (R^2^ ≈ 0.12) reflects the inherently multidimensional nature of medication adherence, underscoring the need for future longitudinal studies incorporating psychiatric symptoms, social support, and intervention-based designs targeting spiritual well-being [35,36].

From a clinical perspective, medication adherence among women with early-stage breast cancer and active parenting responsibilities appears to be associated with psychosocial factors beyond treatment modality or disease severity alone. Parenting stress was identified as a risk marker for poorer adherence, whereas spiritual well-being was associated with more favorable adherence patterns, both independently and within a statistical mediation framework. These findings support the rationale for future longitudinal and interventional studies examining integrated care models that combine side-effect management, behavioral strategies, social support for parenting roles, and meaning- or purpose-centered psychosocial interventions, particularly for vulnerable subgroups [6,21,37].

## 5. Conclusions

Medication adherence in women with early-stage breast cancer who maintain active parenting responsibilities appears to be associated with psychosocial factors beyond clinical and treatment-related characteristics alone. In this cross-sectional study, higher parenting stress was associated with poorer adherence, whereas greater spiritual well-being was associated with more favorable adherence patterns within a statistical mediation framework. These findings are hypothesis-generating and highlight the need for future longitudinal and interventional studies to clarify causal pathways and evaluate psychosocially informed supportive care approaches.

## Figures and Tables

**Table 1:** Baseline Characteristics of the Study Population (n = 432).

Variable	Category	n (%)
Age (years)	Mean ± SD	47.11 ± 8.28
	Median (Min–Max)	47 (24–66)
Marital status	Married	347 (80.3)
	Divorced	50 (11.6)
	Widowed	35 (8.1)
Educational level	Primary school or below	172 (39.8)
	Secondary education	124 (28.7)
	Higher education	136 (31.5)
Income status	Sufficient	229 (53.0)
	Barely sufficient	118 (27.3)
	Insufficient	85 (19.7)
Employment status	Actively working	119 (27.5)
	Not working, with financial support	106 (24.5)
	Not working, without financial support	207 (47.9)
Place of residence	Urban	304 (70.4)
	Semi-urban	66 (15.3)
	Rural	62 (14.4)
Household size	Living alone	20 (4.6)
	2–3 persons	246 (56.9)
	≥4 persons	166 (38.4)
Number of children	1	121 (28.0)
	2	198 (45.8)
	≥3	113 (26.2)
Age of youngest child	0–6 years	59 (13.7)
	7–12 years	133 (30.8)
	13–18 years	101 (23.4)
	≥18 years	139 (32.2)
Primary caregiver for childcare	Self	107 (24.8)
	With spouse	303 (70.1)
	Other family member	22 (5.1)
Daily time spent caring for children	<1 h	81 (18.8)
	1–3 h	122 (28.2)
	4–6 h	108 (25.0)
	≥7 h	121 (28.0)
Companion during treatment visits	Spouse	268 (62.0)
	Child	76 (17.6)
	Other family member	48 (11.1)
	Alone	40 (9.3)
Disease stage	Stage I	125 (28.9)
	Stage II	205 (47.5)
	Stage III	102 (23.6)
Number of medications used	1	207 (47.9)
	2–3	132 (30.6)
	≥4	93 (21.5)

Values are presented as number (percentage) unless otherwise indicated. Percentages are calculated based on the total study population (n = 432).

**Table 2:** Spearman Correlations Between MMAS-6, PSS, and FACIT-Sp-12 Scores (n = 432).

Scale	MMAS-6	PSS	FACIT-Sp-12
MMAS-6	1.00	0.248 **	−0.225 **
PSS	0.248 **	1.00	−0.597 **
FACIT-Sp-12	−0.225 **	−0.597 **	1.00

Values represent Spearman’s rank correlation coefficients (ρ). ** *p* < 0.01, two-tailed. MMAS-6 scores are coded such that higher scores indicate poorer medication adherence.

**Table 3:** Comparison of Mean Rank Scores of MMAS-6, PSS, and FACIT-Sp-12 According to Sociodemographic Characteristics.

Variable	Group (n, %)	MMAS-6 Mean Rank	*p* ^1^	PSS Mean Rank	*p* ^2^	FACIT-Sp-12 Mean Rank	*p* ^3^
**Marital status**	Married (347, 80.3%)	210.4	0.103	210.0	0.036	224.0	0.014
	Divorced (50, 11.6%)	243.3		227.5		202.0	
	Widowed (35, 8.1%)	238.1		265.2		162.3	
**Educational level**	Primary or below (172, 39.8%)	230.3	0.078	215.5	0.157	220.8	0.548
	Secondary (124, 28.7%)	216.0		232.6		206.1	
	Higher education (136, 31.5%)	199.3		202.9		220.5	
**Income status**	Sufficient income (229, 53.0%)	185.9	<0.001	187.7	<0.001	241.2	<0.001
	Barely sufficient (118, 27.3%)	233.2		246.3		179.8	
	Insufficient (85, 19.7%)	275.5		252.5		200.6	
**Employment status**	Actively working (119, 27.5%)	197.9	0.061	185.3	0.004	235.9	0.031
	Not working, with financial support (106, 24.5%)	211.4		220.1		192.1	
	Not working, without financial support (207, 47.9%)	229.7		232.5		217.7	
**Place of residence**	Urban (304, 70.4%)	204.4	<0.001	212.0	<0.001	216.7	0.002
	Semi-urban (66, 15.3%)	218.5		179.3		254.4	
	Rural (62, 14.4%)	273.6		277.7		175.0	
**Number of people living in household**	Living alone (20, 4.6%)	144.8	0.006	316.4	<0.001	98.2	<0.001
	2–3 persons (246, 56.9%)	212.1		203.4		213.7	
	≥4 persons (166, 38.4%)	231.6		223.8		234.7	
**Number of children**	1 child (121, 28.0%)	209.4	0.744	181.6	0.001	222.2	0.719
	2 children (198, 45.8%)	219.4		228.5		211.3	
	≥3 children (113, 26.2%)	218.8		232.7		219.5	

Values are presented as mean ranks; *p* ^1^: Comparison of MMAS-6 scores; *p* ^2^: Comparison of PSS scores; *p* ^3^: Comparison of FACIT-Sp-12 scores; Statistical comparisons were performed using the Kruskal–Wallis test.

**Table 4:** Comparison of Mean Rank Scores of MMAS-6, PSS, and FACIT-Sp-12 According to Clinical Characteristics and Treatment Modalities.

Variable	Group (n, %)	MMAS-6 Mean Rank	*p* ^1^	PSS Mean Rank	*p* ^2^	FACIT-Sp-12 Mean Rank	*p* ^3^
Time since diagnosis	0–6 months (111, 25.7%)	221.5	0.753	209.8	0.638	232.2	0.177
	7–12 months (66, 15.3%)	227.0		228.9		197.9	
	1–3 years (125, 28.9%)	209.9		222.9		204.2	
	≥3 years (130, 30.1%)	213.1		209.7		224.2	
Disease stage	Stage I (125, 28.9%)	213.0	0.124	167.4	<0.001	248.1	0.003
	Stage II (205, 47.5%)	208.2		235.1		200.3	
	Stage III (102, 23.6%)	237.3		239.2		210.1	
Number of medications used	1 (207, 47.9%)	212.0	0.310	179.3	<0.001	251.0	<0.001
	2–3 (132, 30.6%)	229.5		240.8		188.1	
	≥4 (93, 21.5%)	207.9		264.6		179.7	
Primary caregiver for child care	Self (107, 24.8%)	240.8	0.049	235.2	0.197	194.8	0.073
	With spouse (303, 70.1%)	208.9		209.9		221.8	
	Other family member (22, 5.1%)	201.8		215.1		248.3	
Time spent caring for children	<1 h/day (81, 18.8%)	218.5	0.844	260.0	0.002	165.0	<0.001
	1–3 h/day (122, 28.2%)	223.7		221.0		201.1	
	4–6 h/day (108, 25.0%)	210.9		199.0		221.0	
	≥7 h/day (121, 28.0%)	212.7		198.3		262.3	
Chemotherapy	Yes (395, 91.4%)	216.7	0.898	216.2	0.881	219.2	0.138
	No (37, 8.6%)	214.1		219.5		187.4	
Surgery	Yes (420, 97.2%)	215.9	0.548	216.3	0.805	216.1	0.694
	No (12, 2.8%)	237.0		225.3		230.5	
Radiotherapy	Yes (255, 59.0%)	215.9	0.898	230.3	0.006	204.6	0.017
	No (177, 41.0%)	217.4		196.6		233.7	
Hormone therapy	Yes (176, 40.7%)	218.2	0.810	243.0	<0.001	180.1	<0.001
	No (256, 59.3%)	215.4		198.3		241.5	

Data are presented as mean ranks; *p* ^1^: Comparison of MMAS-6 scores; *p* ^2^: Comparison of PSS scores; *p* ^3^: Comparison of FACIT-Sp-12 scores; Statistical comparisons were performed using the Kruskal–Wallis test for variables with more than two groups, and the Mann–Whitney U test for binary variables (yes/no).

**Table 5:** Characteristics Related to Medication Use and Treatment Adherence.

Variable	Value
**Methods Used to Remember Medication Intake, n (%)**	
Alarm or written notes	90 (20.8%)
Support from family members	45 (10.4%)
Self-managed (no external aid)	273 (63.2%)
Frequently forgets to take medication	24 (5.6%)
Factors hindering medication use, n (%)	
Treatment-related side effects	128 (29.6%)
Daily fatigue and stress	72 (16.7%)
Forgetfulness	110 (25.5%)
Distrust toward medication	13 (3.0%)
General difficulty with medication use	109 (25.2%)

Data are presented as numbers (percentages). Participants could report more than one factor affecting medication use.

**Table 6:** Multiple Linear Regression Analysis Predicting MMAS-6 Total Score (n = 432).

Variable	B	SE	β	*t*	*p*
**Constant**	2.434	0.524	–	4.65	<0.001
**PSS**	0.023	0.007	0.180	3.14	0.002
**FACIT-Sp-12**	−0.032	0.009	−0.199	−3.46	0.001

Model statistics: R^2^ = 0.116; Adjusted R^2^ = 0.112; F = 28.10; *p* < 0.001; Dependent variable: MMAS-6 total score (higher scores indicate poorer adherence). B: Unstandardized regression coefficient; SE: Standard error; β: Standardized regression coefficient. *t*: *t*-statistic (B/SE), testing whether the regressioncoefficient differs from zero.

**Table 7:** Hierarchical Linear Regression Analysis Predicting MMAS-6 Total Score (n = 432).

Model	Variable	B	SE	β	*t*	*p*
Model 1	Constant	0.777	0.214	–	3.63	<0.001
	PSS	0.039	0.006	0.302	6.57	<0.001
Model 2	Constant	2.434	0.524	–	4.65	<0.001
	PSS	0.023	0.007	0.180	3.14	0.002
	FACIT-Sp-12	−0.032	0.009	−0.199	−3.46	0.001

Model statistics: Model 1: R^2^ = 0.091; Adjusted R^2^ = 0.089; F(1430) = 43.14; *p* < 0.001; Model 2: R^2^ = 0.116; Adjusted R^2^ = 0.112; ΔR^2^ = 0.025; ΔF(1429) = 11.96; *p* = 0.001. Dependent variable: MMAS-6 total score (higher scores indicate poorer adherence). B: Unstandardized regression coefficient; SE: Standard error; β: Standardized regression coefficient. No multicollinearity was detected in Model 2 (Variance Inflation Factor, VIF = 1.60). *t*: *t*-statistic (B/SE), testing whether the regressioncoefficient differs from zero.

**Table 8:** Mediation Analysis of Parenting Stress (PSS), Spiritual Well-Being (FACIT-Sp-12), and Medication Adherence (MMAS-6) (n = 432).

Path/Effect	B (Unstandardized)	SE	*t*	*p*	95% CI (LLCI, ULCI)
a path: **PSS → FACIT-Sp-12**	−0.484	0.030	−16.06	<0.001	−0.543, −0.425
b path: **FACIT-Sp-12 → MMAS-6 (controlling for PSS)**	−0.032	0.009	−3.46	0.001	−0.050, −0.014
c′ path (Direct effect): **PSS → MMAS-6 (controlling for FACIT-Sp-12)**	0.023	0.007	3.14	0.002	0.009, 0.037
c path (Total effect): **PSS → MMAS-6**	0.039	0.006	6.57	<0.001	0.027, 0.051
Indirect effect (a × b)	0.0155	—	—	—	0.0068, 0.0253

Bootstrap samples = 5000; indirect effects were estimated using bias-corrected bootstrap confidence intervals. MMAS-6 scores were coded such that higher scores indicate poorer medication adherence. B: Unstandardized effect estimate, SE: Standard Error, *t*: *t*-statistic (B/SE), CI: confidence interval, a: Effect of PSS on FACIT-Sp-12, b: Effect of FACIT-Sp-12 on MMAS-6 controlling for PSS, c: Total effect of PSS on MMAS-6, c’: Direct effect of PSS on MMAS-6 controlling for FACIT-Sp-12.

## Data Availability

The data used and analyzed in this study are available from the corresponding author upon reasonable request.

## Abbreviations

The following abbreviations are used in this manuscript:

MMAS-6	6-item Morisky Medication Adherence Scale
PSS	Parenting Stress Scale
FACIT-Sp-12	Functional Assessment of Chronic Illness Therapy–Spiritual Well-Being Scale (12-item)
B	Unstandardized regression coefficient
SE	Standard error
CI	Confidence interval
LLCI	Lower limit confidence interval
ULCI	Upper limit confidence interval
